# Virtual patients versus small-group teaching in the training of oral and maxillofacial surgery: a randomized controlled trial

**DOI:** 10.1186/s12909-019-1887-1

**Published:** 2019-12-04

**Authors:** Lukas B. Seifert, Octavian Socolan, Robert Sader, Miriam Rüsseler, Jasmina Sterz

**Affiliations:** 1 Department of Oral, Cranio-Maxillofacial, and Facial Plastic Surgery, University Hospital Frankfurt, Theodor-Stern-Kai 7, 60590 Frankfurt, Germany; 2 Department of Trauma, Reconstructive and Hand Surgery, University Hospital Frankfurt, Theodor-Stern-Kai 7, 60590 Frankfurt, Germany

**Keywords:** Oral and maxillofacial surgery, Education, Virtual patients, Surgical education, Dental education, Dental students

## Abstract

**Background:**

Computerized virtual patients (VP) have spread into many areas of healthcare delivery and medical education. They provide various advantages like flexibility in pace and space of learning, a high degree of teaching reproducibility and a cost effectiveness. However, the educational benefit of VP as an additive or also as an alternative to traditional teaching formats remains unclear. Moreover, there are no randomized-controlled studies that investigated the use of VP in a dental curriculum. Therefore, this study investigates VP as an alternative to lecturer-led small-group teaching in a curricular, randomized and controlled setting.

**Methods:**

Randomized and controlled cohort study. Four VP cases were created according to previously published design principles and compared with lecturer-led small group teaching (SGT) within the Oral and Maxillofacial Surgery clerkship for dental students at the Department for Cranio-, Oral and Maxillofacial Plastic Surgery, Goethe University, Frankfurt, Germany. Clinical competence was measured prior (T0), directly (T1) and 6 weeks (T2) after the intervention using theoretical tests and a self-assessment questionnaire. Furthermore, VP design was evaluated using a validated toolkit.

**Results:**

Fifty-seven students (VP = 32; SGT = 25) agreed to participate in the study. No competence differences were found at T0 (*p* = 0.56). The VP group outperformed (*p* < .0001) the SGT group at T1. At T2 there was no difference between both groups (*p* = 0.55). Both interventions led to a significant growth in self-assessed competence. The VP group felt better prepared to diagnose and treat real patients and regarded VP cases as a rewarding learning experience.

**Conclusions:**

VP cases are an effective alternative to lecture-led SGT in terms of learning efficacy in the short and long-term as well as self-assessed competence growth and student satisfaction. Furthermore, integrating VP cases within a curricular Oral and Maxillofacial Surgery Clerkship is feasible and leads to substantial growth of clinical competence in undergraduate dental students.

## Background

Since the explosive growth of the Internet in the ‘1990s computerized virtual patients (VP) have spread into many areas of healthcare delivery and education [1]. The recent development of intuitive and easy to use authoring systems as well as an increased awareness for patient safety [2, 3] that imposed economic and ethical restrictions on undergraduate medical education are factors that contributed to the present popularity of VP. Compared to traditional educational formats like small group teaching, VP offer various advantages like flexibility in terms of space and pace for the learner, as well as a high degree of teaching reproducibility since patient cases are standardized and cost effective and a lecturer or an auditorium are no longer necessary [4]. But what are VP exactly?

The present literature offers a variety of definitions and often it remains unclear what kind of educational instrument is meant when referring to the term. Von Zadow et al. define them as “any software that allows case-based training” [5]. This rather general definition is specified by the American Association of Medical Collages (AAMC) that defined VP as “a specific type of computer program that simulates real-life clinical scenarios; learners emulate the roles of health care providers to obtain history, conduct a physical exam, and make diagnostic and therapeutic decisions” [6]. Kononowicz et al. were the first to propose a classification for VP based on a comprehensive literature review and categorization. This working group found that the majority of articles used VP in the form of *Interactive Patient Scenarios* [7].

In the present literature, a number of publications that investigate the effects of VP on the acquisition of clinical reasoning can be found. Triola et al. compared the use VP with live standardized patients to teach diagnostic abilities in sub-diagnostic and acute stress and post-traumatic stress disorders. Using clinical vignettes, they found a significant increase in diagnostic abilities after the intervention. However, their study targeted post-graduate health-care professionals and was carried out in a non-curricular setting. Furthermore, their study group received both VP and live standardized patients, meaning that VP were not examined as an alternative teaching method [8]. Botezatu et al. used three VP cases in the area of cardiology and hematology to teach theoretical knowledge and found a positive early learning effect, but also a better learning retention at a late assessment 4.5 month after the intervention [9]. However, no assessment prior to the intervention was carried out. Kerfoot et al. came to similar results in their study that examined the use of 4 VP cases to teach clinical reasoning in the field of urology to undergraduate medical students [10]. Even though, their study was carried out in a curricular, multi-institutional setting with 210 study participants, it did not investigate the use of VP as an alternative teaching method since all students received standard lectures within their urology apprenticeship. For dental education in particular, Zary et al. evaluated student’s perception and satisfaction for a newly web-based VP platform. They determined a high level of satisfaction among the students. However, their study did not investigate an objective learning progress using validated instruments [11]. This also applies to the study of Gerhardt-Szep et al. that comprehensively described the design and implementation of 5 VP cases in undergraduate dental education and found high student satisfaction with the teaching format, but did not assess a knowledge increase after the intervention [12]. Regarding practical skills training there are several studies that have shown beneficial effects of VP for emergency medicine and basic life support [13–15] . Kononovicz et al. showed that students who were trained with a voluntary VP module showed better overall BLS-AED action skills compared to a control group. Lehman et al. showed that VP are an effective addition to skills laboratory training and lead to significant improvement in pediatric basic life support [16] and student satisfaction [15].

Despite this large body of literature, the educational benefit of VP is still being debated. In particular, it remains unclear whether VP have a positive effect on the acquisition of skills and clinical reasoning competencies as an additive [17] or also as an alternative [18] to traditional teaching formats. Unfortunately, only a few studies were carried in a controlled and curricular “in vivo” setting or focus on the long-term retention of the knowledge acquired [8, 9]. For Oral and Maxillofacial Surgery however, we were not able to find any study that met the aforementioned standards even though it is reported that VP are frequently (range 15 to 63%) used among dental schools [19, 20]. Most studies rather describe the technical development and feasibility of VP case creation [21] or investigate students’ perception and self-assessed learning progress using VP cases [11, 12, 22].

Therefore, the present preliminary study examines the use of VP in the short and long-term acquisition of theoretical knowledge and clinical reasoning skills in Oral and Maxillofacial Surgery compared to lecturer-led Small Group Teaching (SGT) in a controlled and curricular setting using validated instruments. The underlying question of our study was if VP Learning was equally effective in the acquisition of the above mentioned skills compared to standardized lecturer-led SGT.

## Methods

### Study design and participants

Fifty-seven (female *n* = 39; male *n* = 18) 4th year dentistry students in a five-year program without previous experience in the field of Oral and Maxillofacial Surgery were assigned randomly in a VP and a SGT group which was regarded as a control group. Participation in the study was voluntary and took place after written informed consent, which was revocable at any time. Students were blinded during both of the instructional approaches as well as affiliation to any study group. Basic data regarding student age, sex, and duration of study were collected using a questionnaire. The study was reviewed by the ethical committee of the University Hospital of Frankfurt (Johann-Wolfgang Goethe University) and it was stated, that no further approval was required.

### Assignment of the students to the instructional approaches

The assignment of students to one of the learning groups with a maximum of 6 students per week occurred prior to the Oral and Maxillofacial Surgery apprenticeship independent of the authors and independent of study participation by the deanery. The assignment of the learning groups in the study to the VP instructional approach and the traditional SGT approach took place alternately within the 10 week span of the apprenticeship.

### Study protocol

The study was carried out within the Oral and Maxillofacial Surgery apprenticeship for dentistry students, which includes a five-day rotation through every section of the Department of Oral, Cranio-Maxillofacial and Facial Plastic Surgery, i.e. the operative room, the outpatient clinic or the emergency department. Before starting their rotation, students have to complete a *practical skills training*. The aim was to give dentistry students a short overview of the most common consultation reasons in Oral and Maxillofacial Surgery and prepare them for the upcoming clinic rotation. It is divided into a theoretical part (240 min) in which the study took place in the morning and a practical skills training (240 min) in the afternoon. Trained practical skills include performing a structured facial examination, placing a venous catheter and an Ernst ligature. Lessons were held in small groups ranging from four to six students [23].

### Virtual patient group

Before starting the intervention, students were instructed in the usage of the e-learning platform “*Lernbar*” [24] by trained tutors. Lernbar is offered by *Studiumdigitale* which represents Goethe University’s main e-learning institution [25]. Prior to the intervention, four interactive VP cases (case access on request) were created with the Lernbar author system according to the 10 design principles for VP cases namely being relevant, possess an appropriate level of difficulty, being highly interactive and rich in specific feedback, making optimal use of media, focus on relevant learning points and offer recapitulation. Furthermore, each VP case provided an authentic web-based interface and contained questions and explanations tailored to the clinical reasoning process [26]. Each VP case equals a main theoretical topic of the practical skills training in terms of content and learning objectives; the topics include the clinical management of common traumatological, infectiological and oncological Oral and Maxillofacial Surgery consultations.

Each case was enriched with numerous elements like drag-and-drop, drop-down-menus and videoclips to create a multimedia e-learning environment (Additional files 2 and 3). Cases were constructed in a linear and non-dichotomous way, but students could freely navigate back to previous case slides to look up relevant findings within the cases. To compensate for the missing interaction with a teaching physician on-site at the clinic, diagnostics and treatment options were provided in additional text-boxes (glossary), and multiple-choice questions were used. Correct answers were rewarded with motivational feedback and further information on the case, while wrong answers led to constructive feedback and detailed explanations regarding the various choices. Each case was developed by three medical education experts of whom two hold a masters degree and one is a PhD for medical education. The first author of this study was responsible for the correctness of the case content. After the initial case development the cases were piloted by five dental students and five employees of the Department of Oral, Cranio-Maxillofacial, and Facial Plastic Surgery, University Hospital Frankfurt. After piloting the cases were slightly adjusted in content and technical issues i.e. non-functioning answer choices within multiple-choice questions were corrected. The cases only differed slightly in length (38 to 40 slides per case).

Following the instruction, students had 240 min to complete all four VP cases. In order to achieve a better comparability between both groups, cases had to be completed in the same order and in the same seminar room in which the SGT group was taught. Students were allowed to exchange ideas and share information while working on VP cases just like SGT students.

### Small group teaching group

Students of SGT group were taught by two previously trained teaching physicians using standardized (Microsoft Power-Point®, Microsoft Corporation, Redmond, WA) presentations on the aforementioned learning objectives. Each presentation provided key information on the prevalence, diagnostic approach, management of emergencies, surgical treatment options and rehabilitation of common Oral and Maxillofacial Surgery consultations and was built as a patient casuistry. The presentations were held in a seminar setting with groups of four to six students. Students were guided through the presentations but were encouraged to actively participate and frequently ask questions during the presentations. Moreover, multiple-choice questions identical to the questions used in the VP-cases were integrated and answers options were discussed orally within the SGT group with direct feedback from the teaching physician. The learning objectives were identical to the VP cases. Both teaching physicians received a script on the learning objectives and were provided with detailed guidelines and instructions. SGT was performed in the same seminar room and during the same time (240 min) as the VP group.

### Performance measurement

To measure the learning success and evaluate the VP design, the following qualitative and quantitative tests were used:

1. A theoretical test (Additional file 1) was used prior, directly after the teaching unit and 6 weeks (range: four to 8 weeks) after the intervention as a retention test within a non-graded Oral and Maxillofacial Surgery examination. The test was validated by three Oral and Maxillofacial Surgery experts and it was composed of 20 multiple-choice questions that were randomly taken out of a pool of 100 multiple-choice questions from previous theoretical examinations within the CMF clerkship for dental students over the last five semesters and that cover the preassigned learning objective in equal parts. This way of testing should prevent students from memorizing the questions between the testing times. To calculate the total of test points a bonus-malus-system was applied.
2. A self-assessment questionnaire (Table 1) which was composed of 13 questions covering the preassigned learning objectives. Students were asked to rate their Oral and Maxillofacial Surgery knowledge and competencies on a 6-point Likert scale ranging from “1= very good “to “6 = insufficient” prior and directly after the intervention.
3. Form 1 of the Virtual patient design and curricular integration evaluation toolkit (Table 2) developed by Huwendiek and de Leng [27] was used to evaluate the design of VP cases after case completion. The questionnaire was composed of 15 statements that students of the VP group were asked to evaluate using a 5-point Likert scale ranging from “1 = strongly disagree” to “6 = strongly agree”.

**Table 1:** Self-assessment prior (T0) and post (T1) intervention

Item		T0	T1	*p*-Value
Anatomy of the cranio-facial area	SGT	3.36	3.08	0.075 (M)
	VP	3.24	3.21	0.833 (M)
	*p*-Value	0.49	0.58	
Clinical signs of a mandibular fracture	SGT	4	2.92	**<.001** (M)
	VP	3.94	2.91	**<.0001** (M)
	*p*-Value	0.94	0.96	
Diagnostic approach of mandibular fractures	SGT	4.24	2.88	**<.0001** (M)
	VP	4.35	3.03	**<.0001** (M)
	*p*-Value	0.37	0.54	
Therapy of mandibular fractures	SGT	4.2	2.92	**<.0001** (M)
	VP	4.26	3.03	**<.0001** (M)
	*p*-Value	0.58	0.60	
Diagnostic approach of midfacial fractures	SGT	4.52	3.08	**<.0001** (M)
	VP	4.68	3.12	**<.0001** (M)
	*p*-Value	0.303	0.936	
Therapy of midfacial fractures	SGT	4.6	3.12	**<.0001** (M)
	VP	4.62	3.29	**<.0001** (M)
	*p*-Value	0.624	0.502	
Clinical signs of oro-facial infections	SGT	4.04	3.08	**<.001** (M)
	VP	4.06	2.74	**<.0001** (M)
	*p*-Value	0.904	0.118	
Diagnostic approach of oro-facial infections	SGT	4.32	3.2	**<.001** (M)
	VP	4.41	2.97	**<.0001** (M)
	*p*-Value	0.659	0.271	
Therapy of oro-facial infections	SGT	4.28	3.04	**<.001** (M)
	VP	4.50	3.00	**<.0001** (M)
	*p*-Value	0.406	0.771	
Risk factors for the development of malignant head and neck tumors	SGT	3.88	2.96	**<.001** (M)
	VP	3.85	2.94	**<.001** (M)
	*p*-Value	0.992	0.880	
Clinical signs of malignant head and neck tumours	SGT	4.16	3	**<.001** (M)
	VP	4.24	2.97	**<.0001** (M)
	*p*-Value	0.631	0.825	
Diagnostic approach of malignant head and neck tumours	SGT	4.16	3.12	**<.001**
	VP	4.32	3.15	**<.0001**
	*p*-Value	0.555	0.857	
Therapy of malignant head and neck tumours	SGT	4.28	3.28	**<.001**
	VP	4.56	3.15	**<.0001**
	*p*-Value	0.347	0.610	

Data are presented as Mean. Participants rated their knowledge using a 6-point likert scale ranging from “1 = very good “to “6 = insufficient”

(M) = Mann-Whitney-White U test for ordinally distributed data

(T0) = prio to the intervention, (T1) = directly after the intervention

Significant results were marked in boldface

**Table 2:** Results from the *Virtual patient design and curricular integration evaluation toolkit* developed by Huwendiek and de Leng

	1	2	3	4	5	6	Mean	SD
**Authenticity of patient encounter and the consultation**								
While working on this case, I felt I had to make the same decisions a doctor would make in real life.	3	8	8	10	0	0	3.12	1.17
While working on this case, I felt I were the doctor caring for this patient.	1	11	13	8	0	0	2.85	0.82
**Professional approach in the consultation**								
While working through this case, I was actively engaged in gathering the information (e.g., history questions, physical exams, lab tests) I needed, to characterize the patient’s problem.	4	6	10	10	3	0	3.06	1.15
While working through this case, I was actively engaged in revising my initial image of the patient’s problem as new information became available.	2	4	10	11	5	1	3.48	1.16
While working through this case, I was actively engaged in creating a short summary of the patient’s problem using medical terms.	6	3	7	8	9	0	3.33	1.43
While working through this case, I was actively engaged in thinking about which findings supported or refuted each diagnosis in my differential diagnosis.	1	3	10	13	5	1	3.64	1.04
**Coaching during consultation**								
I felt that the case was at the appropriate level of difficulty for my level of training.	0	5	9	14	5	0	3.58	0.92
The questions I was asked while working through this case were helpful in enhancing my diagnostic reasoning in this case.	0	1	3	19	10	0	4.15	0.70
The feedback I received was helpful in enhancing my diagnostic reasoning in this case.	0	1	3	17	12	0	4.21	0.73
**Learning effect of consultation**								
After completing this case, I feel better prepared to confirm a diagnosis and exclude differential diagnoses in a real life patient with this complaint.	0	0	6	12	15	0	4.27	0.75
After completing this case I feel better prepared to care for a real life patient with this complaint.	0	1	8	15	8	1	4.00	0.85
**Overall judgment of case workup**								
Overall, working through this case was a worthwhile learning experience.	0	0	2	10	21	0	4.58	0.6
**Special strengths of the case:** Video footage illustrated investigation very closely; Individual speed; Well structured, detaileled; interesting cases; multimedia presentation, Clear structure; real pictures; Detailed explanation of the basics; multiple editing possible, understandable, supported with images; good portioning of content in “learning packages”								
Special weaknesses of the case: In the answers, the individual feedback should be shown directly; Staging and CT findings overstraining; processing time too short								

Significant results were marked in boldface

### Statistical analysis

Microsoft Office 2016 (Microsoft Office 2007,© Microsoft Corporation, Redmond, USA) for Mac and SPSS Statistics version 19 (IBM, Armonk, USA) were used for the statistical analysis and graphical display of data.

To test for a normal distribution of the data the Shapiro-Wilks-Test was used. Since the test results of both groups were not normally distributed at all times (T0 W = 0.93; T1 W = 0.94; T2 W = 0.91) the Mann-*Whitney-U-Test for non-parametric data* was used to test for significant differences in learning success in the intergroup comparison at T0 to T2. To test for performance differences within the respective groups at different times, the Friedman Test for Repeated-Measures with Bonferroni correction was used. To reduce statistical biases, parwise comparisons between T0, T1 and T2 were additionally calculated. To test for significant differences in the self-assessed competence the Mann-*Whitney-U-Test for non-parametric data* was used since this data was ordinally distributed. To test for an unequal gender distribution an unpaired t-test was used.

Furthermore, effect sizes were calculated for T0 to T2 using Cohens d. Cohen’s d is defined as the difference between two means divided by a standard deviation for the data resulting in an unitless value that helps to interpret the effect size of observed results and hence the statistical power of a study. For most types of effect sizes, a larger absolute value indicates a stronger effect. Since the sample size (*n* = 57) of our study was relatively small Cohen’s d was used as an additional control test since prior studies have shown significant test results alone are not sufficient to interpret data and draw conlusion from this data [28].

The statistical analyses of the data was carried out by a doctoral student who was blinded regarding the assignment of the data to respective learning groups.

### Sample size estimation

Based on prior examination results from the years before the intervention we estimated an average student performance of 70% with a standard deviation of 10% in the theoretical test. Based on the following paramenter (Mean VP = 53, Mean SGT = 50, SD = 5, alpha = 80%, beta = 20%) a sample size of 88 was calculated.

## Results

### Study participation and gender distribution

Thirty-two VP students (f = 20; m = 12; average age = 25) and 25 SGT students (f = 19; m = 6; average age = 25) took part in the study. There was no significant (*p* = 0.28) difference in gender distribution between both groups. One student was excluded due to a completed degree in medical studies. Both teaching interventions could be carried out in a curricular setting in the given timeframe without any complications.

### Outcome measures

#### Theoretical test

Prior to the intervention both groups nearly achieved the same results in the theoretical test (Table 3). The VP group achieved 30.2 out of 76 possible points (SD = 8.7) while the SGT group achieved 30.7 points (SD = 6.2) respectively. There was no significant difference between both groups (*p* = 0.56; d = 0.06). After the intervention both groups significantly increased their performance (*p* < 0.0001) compared to the pretest. However, there was a highly significant (*p* < 0.0001; d = 2.41) performance difference in favor of the VP group which doubled its test scores with 60.8 points (SD = 9.6) and showed a stronger effect size (d^VP^ = 3.3 vs. d^SGT^ = 1.42) than the SGT group which achieved 40.3 points (SD = 7.2). In the formative Oral and Maxillofacial Surgery examination 6 weeks after the intervention no significant difference (*p* = 0.56; d = 0.1) between the groups could be found. With 65.2 (SD = 7.6) points the VP group showed a good long-term knowledge retention. The SGT managed to significantly (*p* < 0.001; d = 3.3) improve its test scores with 64.4 points (SD = 7.3).

**Table 3:** Average scores obtained by the respective groups in the theoretical tests (76 possible points) and corresponding significance levels

	T0	T1	T2	*p*-Value T0-T1	*p*-Value T1-T2	*p*- Value T0-T2
SGT	30.68 (+ 6,2)	40.32 (+ 7,2)	64.44 (+ 7,3)	**0.00003** (F)	**< .00001** (F)	**< 0.00001** (F)
VP	30.18 (+ 8,7)	60.79 (+ 9,6)	65.24 (+ 7,6)	**< 0.00001**(F)	**0.05** (F)	**< 0.00001**(F)
*p*- Value	0.564 (M)	**< 0.001** (M)	0.565 (M)			
Effect size (Cohens *d*)	0.066	2.412	0.107			

Data are presented as Mean + SD

(F) = Friedman test for repeated test measures

(M) = Mann-Whitney-White U test for ordinally distributed data

(T0) = prio to the intervention, (T1) = directly after the intervention, (T2) = six weeks after the intervention

Significant results were marked in boldface

#### Self-assessment questionnaire

In the self-assessment questionnaire both the VP group and the SGT group rated their competence regarding the predetermined learning objective significantly higher than before the intervention (See Table 1). No significant differences in self-assessed competence could be found after the intervention.

#### Virtual patient design evaluation

Overall, students felt better prepared to diagnose (median = 4; average = 4.3) and treat (median = 4; average = 4.0) real patients after completing the four VP cases (See Table 2). Students felt that working on the VP cases was a rewarding learning experience (median = 5; average = 4.6) and found the degree of difficulty appropriate (median = 4; average = 3.8). The direct feedback that was given within the VP cases was felt to be sufficient (median = 4; average = 4.2). Students also found that case completion was beneficial regarding their clinical reasoning competences (median = 4; average = 4.1). In particular, the detailed structure, the multi-media environment, the individual learning pace and the option to repeatedly work on cases was commended. However, students only partially found that working on the VP cases felt like making real life clinical decisions (median = 3; average = 3.1). Also, they missed direct interaction with a lecturer to clarify open-ended questions and found the given time for case completion (240 min) to be too short.

## Discussion

This study was conducted to examine the use of VP cases in the short and long-term acquisition of theoretical knowledge and clinical reasoning in a controlled and curricular “in vivo” setting as an alternative to lecture-led SGT within an Oral and Maxillofacial Surgery clerkship. A second goal was to compare self-assessed knowledge increase and evaluate VP case design using validated measures. Overall, our results revealed significant differences between the teaching formats with regard to short-term increase in theoretical knowledge. No significant differences were found for long-term knowledge retention. The self-assessed learning progress was perceived to be equal to SGT seminars. Students of the VP group evaluated the design, quality, content and comprehensibility of VP seminars as good and rated their clinical reasoning competence significantly better than before the intervention.

### Outcome measures

#### Theoretical test

The results of the theoretical test prior to the teaching intervention indicate similar prerequisites regarding theoretical knowledge from the Oral and Maxillofacial Surgery spectrum and hence a good comparability between both groups.

Both teaching interventions were clearly beneficial for the students regarding their theoretical Oral and Maxillofacial Surgery knowledge since there were significant performance increases in the theoretical test directly after the teaching intervention. However, with an effect size twice as high (d^VP^ = 3.3 vs. d^SGT^ = 1.42) the VP group seemed to have profited more from working on the VP cases than the SGT group. A reason for this big performance gap might be that students of the VP group were able to work on VP cases at their own pace of learning. Flexibility in terms of time and pace of learning is one of the main advantages of VP. It shifts the learning experience from a teacher-centered to a more learner-centered perspective and has already been described in previous studies [17, 29]. This might have led to a deeper understanding and knowledge retention in the VP group. However, these results must be interpreted with caution since students were already accustomed to answer multiple-choice questions similar to the theoretical test questions while working on the VP cases. This phenomenon, also known as testing effect, might have influenced our results and has been thoroughly described by Kromann et al. [30].

In the long-term theoretical test both groups were able to increase their performance compared to the second assessment directly after the intervention. The VP group showed an enhanced performance while the SGT group was able to significantly increase its performance up to the level of the VP group. A reason for this unusual high increase in performance might be due to the prior assessment in which the SGT group was confronted with its inferior performance and thus might have been motivated to perform better. Another possible reason might be that students wanted to perform well in the Oral and Maxillofacial Surgery examination 6 weeks after the intervention even though it was carried out in a formative way. Previous studies have found a clear correlation between the type of assessment and resulting student performance. This was found to be particularly true for summative assessments, which tend to show an increase in student performance regardless of the prior training format [31]. Even though in our study the long-term theoretical test was carried out as a formative examination, the desire of students from both groups to perform well may have influenced our results.

The unequal gender distribution between the VP and SGT group, even though it was not statistically significant, must be considered when interpreting the results of this study since prior studies have shown significant performance differences between female and male students in medical examinations [32].

#### Student’s self-assessment and VP design evaluation

Previous studies found that self-assessed competence often significantly diverges from objectively measured competence levels [33–35]. Nevertheless, being aware of one’s own competencies and limitations is crucial for taking the first steps as a doctor. Interestingly, there was no significant difference in self-assessed competence levels after the intervention, even though the VP group clearly outperformed the SGT group in the theoretical test. A possible reason for this difference might be the lack of direct oral feedback in the VP cases. Feedback, as a direct response regarding a student’s learning success, plays a crucial part in self-assessed competence. Various studies [36–38] have highlighted the importance of structured feedback and found that the way feedback is given has a significant influence on the learning outcome. In a comprehensive literature review Lechermeier and Fassnacht found that “feedback is most effective when provided by a source who disposes over high status and expertise” [39].

For VP case design in particular, Huwendiek and his working group identified expert feedback as one of ten integral parts that lead to a high teaching efficacy of VP cases [25]. The results of the VP design evaluation reflect this hypothesis. Many students reported a lack of motivation while working due to the missing direct interaction with a lecturer to clarify open-ended questions which could not be answered by the information or feedback given within the VP case. This was regarded as a specific weakness of VP cases by many students and could have contributed to a lower self-assessment after case completion.

### Limitations and strengths

There are some limitations to this study that have to be considered when interpreting the results shown. First, the sample size (*n* = 57 students) is a limitation to the statistical power of this preliminary study. As mentioned previously, the results of the theoretical short and long-term test might have been influenced by the testing effect (short-term) and by the desire of both study groups to perform well in the Oral and Maxillofacial Surgery examination 6 weeks after course completion (long-term), even if this examination was carried out in a formative way. This might have led to false positive results in the theoretical test. Each theoretical test was composed of 20 different (randomized) questions. Due to the curricular framework of the study and the specificity of the predefined learning objectives those tests were only validated by experts without measuring a retest reliability which is a limitation to this study.

There are also several strengths to this study. Compared to other studies, the present study was randomized and controlled to assess the use of VP as an alternative to standard teaching in multiple levels. Another strength is the curricular “in vivo” study design that demonstrates the feasibility of VP as an alternative to another teaching intervention within a curricular apprenticeship which is reinforced by reaching a 100% participation rate and an entire cross-section of an 8th semester at an accredited dental school. Furthermore, the knowledge assessment within three points in time over a six-week span gives a comprehensive overview over the learning progress for both types of teaching interventions.

Future studies have to investigate whether the results obtained from this study can be transferred to other subjects and faculties.

## Conclusion

The results of the present preliminary study show that VP cases are an effective alternative to lecture-led SGT in terms of learning efficacy in the short and long-term as well as self-assessed competence growth and student satisfaction. Furthermore, we were able to show that integrating VP cases is feasible within a curricular Oral and Maxillofacial Surgery Clerkship and leads to substantial growth of clinical competence in undergraduate dental students.

Future studies should examine the actual cost effectiveness provided by the use of VP and compare different forms of case designs and especially how to effectively implement expert feedback into VP cases since these questions remain unclear but are of great importance in this growing field of educational technology.

## Supplementary information

**Additional file 1.** Self-assessment questionnaire.

**Additional file 2.** VP case example: Traumatology case 2.

**Additional file 3.** VP case example: Traumatology case 1.

## Data Availability

The datasets used and/or analysed during the current study are available from the corresponding author on reasonable request.

## Abbreviations

OSCE: Objective Structured Clinical Examination
SGT: Small Group Teaching
VP: Virtual Patients
