# Supplementary material for: Virtual patients versus small-group teaching in the training of oral and maxillofacial surgery: a randomized controlled trial
Source: BMC Med Educ. 2019 Dec 4;19:454. doi: 10.1186/s12909-019-1887-1 (PMC6894350; doi:10.1186/s12909-019-1887-1)
Supplement: Supplementary file 1 — Additional file 1. Self-assessment questionnaire. [file 12909_2019_1887_MOESM1_ESM.pdf]

Supplement 1: Self-assessment questionnaire

Please assess your knowledge of the following items by using the scholar grading system:

1) Very good
2) Good
3) Satisfactory
4) Sufficient
5) Poor
6) Insufficient

Anatomy of the cranium
Clinical signs of the mandibular fracture (MF)
Diagnosis of mandibular fractures
Therapy of mandibular fractures
Diagnosis of midfacial fractures
Therapy of midfacial fractures
Clinical signs of oro-facial infection
Diagnosis of oro-facial infection
Therapy of oro-facial infection
Risk factors for the deveopment of malignant head and neck tumours
Clinical signs of malignant head and neck tumours
Diagnosis of malignant head and neck tumours

Therapy of malignant head and neck tumors

1) Which of the following statements holds true?

- o The bony walls of the orbita are formed by the following bones: maxillary, zygomatic, sphenoid, ethmoid, nasal, frontal, palatine and lacrimal.
- o The infraorbital nerve runs along the floor of the orbit and passes through the infraorbital canal to emerge from the zygomatic bone on the face.
- o The zygomatic bone is topographically closely related to the temporal muscle, coronoid process of the mandible and the frontal, maxillary and temporal bones.
- o The lateral and superior walls of the orbit are the most likely to fracture due to their delicate structure.

2) Check all the statements that apply

- o Loss of vision can be a sign of compression of the optic nerve, as an effect of e.g. retrobulbar hematoma.
- o Decreased eye movement can be caused by an entrapment of orbital content in the fracture gap.
- o Posterior displacement of the eyeball (enophtalmos) can remain unnoticed if the eyelid is swollen.
- o The infraorbital nerve runs through the lower level of the orbit and the maxillary sinus and eventually emerges on the surface of the skull through the infraorbital foramen.

3) Check all that apply.

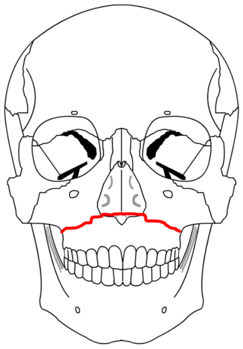

I, RosarioVanTulpe [CC BY-SA 3.0]

- The fracture line corresponds with that of a LeFort III fracture.

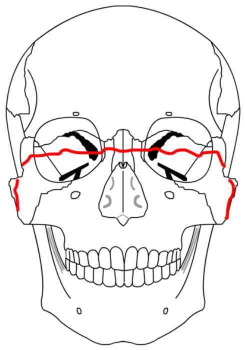

I, RosarioVanTulpe [CC BY-SA 3.0]

- The fracture line is consistent with that of a pyramidal fracture of the midface.

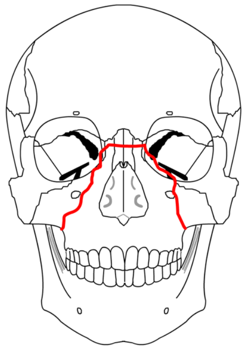

I, RosarioVanTulpe [CC BY-SA 3.0]

- The fracture line corresponds with that of a LeFort I fracture.

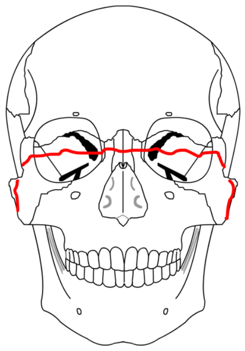

I, RosarioVanTulpe [CC BY-SA 3.0]

- The depicted fracture line runs through the lateral orbital wall, the orbital floor and includes frontonasal structures and can thus be classified as a LeFort III fracture.

4) Which of the following statements holds true?

- 1 represents a branch of the maxillary artery

- o 2 is supplied by the common carotid with the ophthalmic artery as an intermediary
- o 3 marks a predilection site for epistaxis
- o 4 represents the anterior ethmoidal artery
- o None of the above

5) Which of the following statements holds true?

- o Facial fractures are to be treated surgically immediately, as delays can cause cosmetic defects
- o Sensitivity disorders in the area of the infraorbital nerve represent an indication for surgery, since the nerve can be pinched by dislocated bone fragments
- o The interval between 7 and 10 days is considered as optimal for surgical treatment of midfacial fractures.
- o Soft tissue injury should not be used as surgical approaches, as this may cause cosmetic defects

6) Information about which of the following risk factors/ symptoms is relevant with regard to oral squamous cell carcinoma?

- o Alcohol consumption
- o Tobacco consumption
- o Family history of cancer
- o Fever, night sweats, weight loss during the past 6 months
- o Infection with HPV type 16 and 18

7) Which of the following diagnostic features can be used to for grading and staging of oral malignancies?

- o Biopsy
- o MRI of the head and neck
- o Thoracoabdominal CT scan
- o Bone scintigraphy
- o Sonography of cervical lymph nodes

8) A biopsy and an MRI scan with a contrast agent revealed the following findings:
1) Primary tumor located in the right half of the tongue, measuring 3,4 cm at its widest.
2) No midline crossing. 3)No bone invasion. 4)Metastasis of ipsilateral cervical lymph node of the jugulodigastric group with central necrosis.

Which of the following holds true?

- o With regard to image a. the pirmary tumor can be classified as T3
- o Presuming the lymph node metastasis in images b and c is the only one, the regional lymph node status could be classified as N1 or N2a
- o The lymph node metastasis has caused a ventral displacement of the carotid sheath.
- o Image a. and b. are sagittal sections while image c is a transversal section

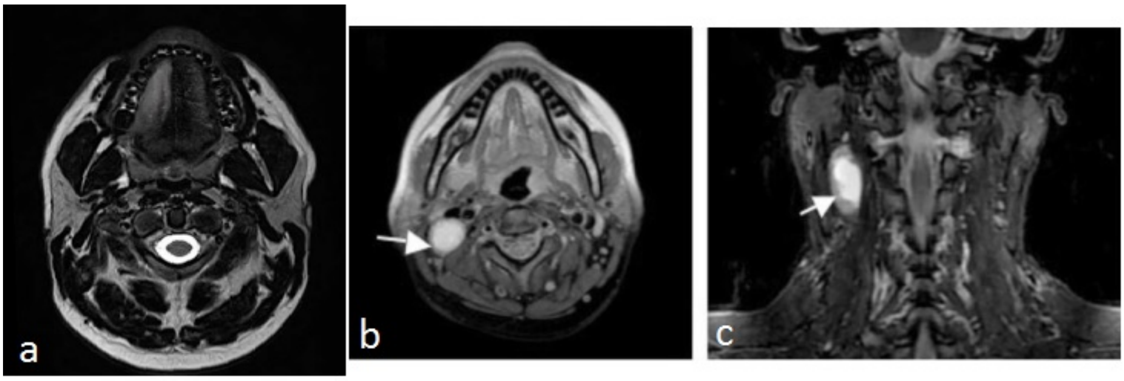

9) Check all that apply
- o Radical neck dissection encompasses the surgical removal of the sternocleidomastoid muscle and the ipsilateral internal jugular vein amongst other cervical structures.
- o In the case of a negative regional lymph node status the incidence of occult lymph node metastasis can be as high as 20 to 40%
- o Primary cervical metastases are often diagnosed in the jugulodigastric region.
- o A surgical margin of 1-3 mm is considered as a safe margin

10) Check all that apply
- o Neoadjuvant radiotherapy is performed prior tot he surgical intervention
- o The oral squamous cell carcinoma is immune to cytostatic agents because of its characteristic point mutation of the p53 gene.
- o The indication for an adjuvant radiotherapy is given after every R0-Resection
- o Xerostomia is a frequent adverse effect of radiotherapy

11) Name the five cardinal clinical signs of inflammation:

12) Thrombophlebitis can occur as a complication of abscesses in the facial region, that can lead to a thrombosis of the cavernous sinus. Which of the following pathways can lead to a retrograde spread of bacteria from facial to intracranial areas?

- o Cavernous sinus> V. ophtalmica > V. angularis > V. infraorbitalis > V. facialis
- o V. facialis > V. ophtalmica > V. angularis > V. infraorbitalis > Cavernous sinus
- o V. facialis > V. angularis > V. infraorbitalis > V. ophtalmica > Cavernous sinus
- o V. infraorbitalis > V. facialis > V. angularis > Cavernous sinus > V. ophtalmica
- o V. facialis > V. infraorbitalis > V. angularis > V. ophtalmica > Cavernous sinus
- o V. angularis > V. ophtalmica > V. facialis > V. infraorbitalis > Cavernous sinus

13) Place the following therapeutical steps in the correct order:
1) Postoperative stay.
2) Premedication
3) Incision and drainage
4) Obtaining informed consent

5) Infectious source control

- o 1 >2 >3 >4 > 5
- o 2 >4 >1 >3 > 5
- o 4 >2 >3 >5 > 1
- o 4 >2 >3 >1 > 5
- o 4 >2 >5 >3 > 1

14) Check all that apply

pH	O2	CO2	BE	Hb	Lc	Tc	Q	INR	Tz1	Tz2	Fibrin	CRP	PCT	Gluc	Na(P)	K(P)	Crea	Phos	CK	TropT	BNP	ASAT	Bili
				126	7.2	282																	
												41.5			141	3.7	64	1	144			22	4
												73.2			139	3.7	61	0.9	129			27	6
				118	7.5	265																	
				126	10.6	265																	
												111			141	3.9	64	1	132			23	13
				141	14.8	301	86	1.1															
												89		5.7	140	4.2	62	1.3	151			24	17

\- +

- o During the last two measurements CRP levels were elevated, fact which indicates a resurgene of the infection
- o During the last two measurements CRP levels were elevated, which is normal considering the 48h delayed response of CRP plasma levels to infection
- o During the last two measurements CRP levels were elevated, which is normal considering the 12 to 24h delayed response of CRP plasma levels to infection
- o The number of leucocytes drops down to a physiological level

15) Check all that apply

- o
- o
- o A pyramidal disjunction of the maxilla can be classified as a Le Fort I pattern fracture
- o A frequent fracture pattern of the mandible is a combined paramedian and contralateral collum fracture

16) The panoramic radiograph is showing the following fracture(s) (check all that apply)

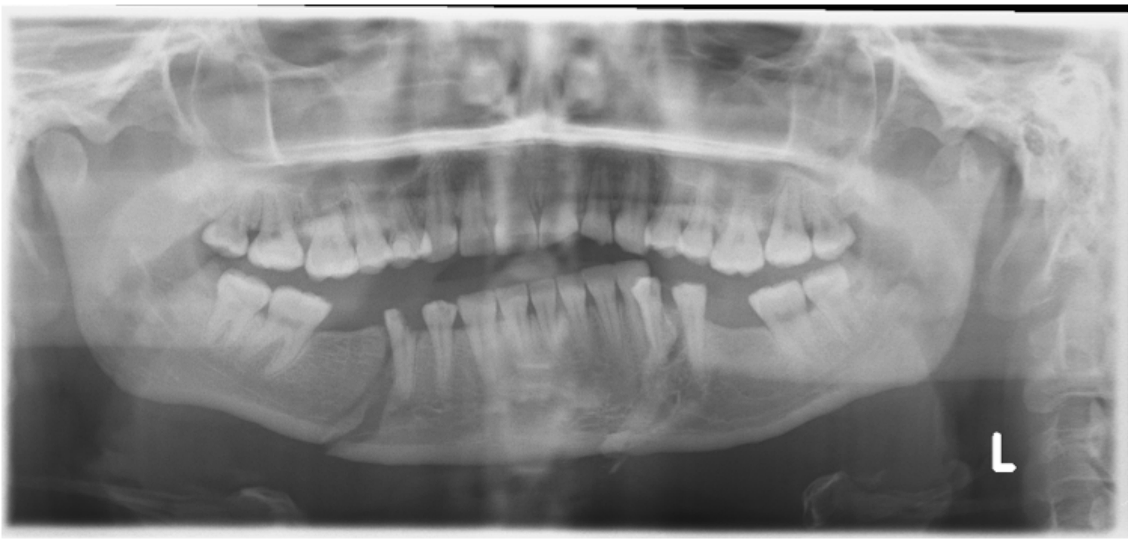

- Right mental fracture
- Left paramedian fracture
- Symphyseal fracture
- Fracture of the ramus
- Fracture of the caput mandibulae
- Right angle fracture
- Right paramedian fracture

17) Assumed the following patient is having trouble swallowing and breathing and is not capable anymore to stretch his or her tongue out, which of the following anatomical deficiencies might be causal?

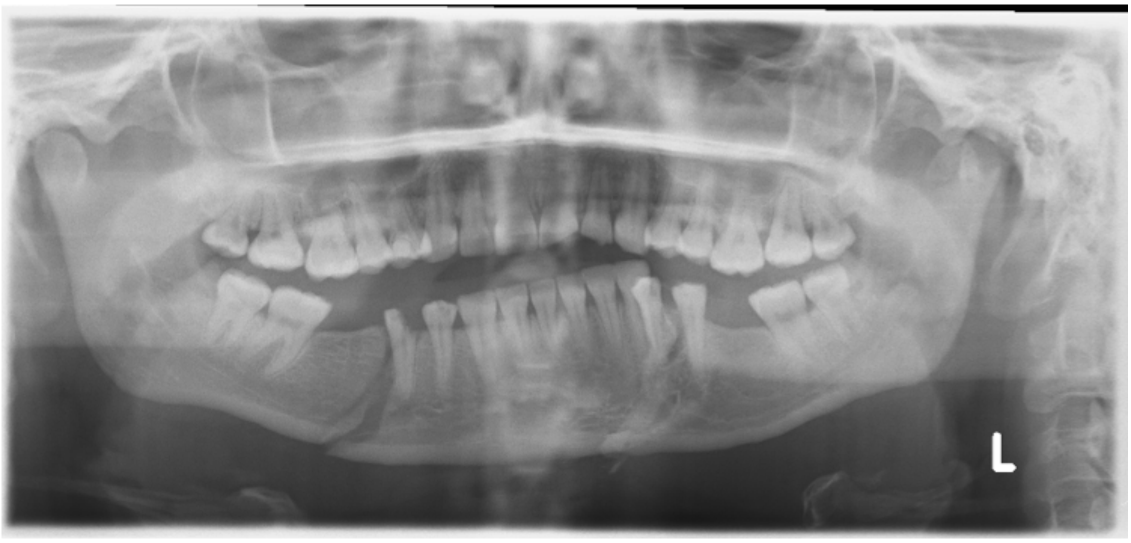

- Suprahyoid muscles have lost their attachment to the mandible and are exerting pulling the hyoid bone cranially
- Given the bilateral paramedian fracture pattern, the suprahyoid muscles lose their attachment to the mandible and can cause a life threatening airway obstruction
- Lesions of the Hypoglossal nerve are a frequent cause of mandibular fracture because of their proximity to the mandibular bone

- A traumatically caused hematoma of the oropharynx can cause an obstruction of the airways

18) Check all that apply

- Fractured teeth have to be included in the figure of eight ligation in mandibular fractures
- The Schuchardt splint is indicated for simple, non displaced fractures
- The intermaxillary fixation is well suited for the conservative therapy of collum fractures
- Drawbacks of the arch bar and ligature of eight are trauma of the periodontium
