# Supplementary material for: Virtual patients versus small-group teaching in the training of oral and maxillofacial surgery: a randomized controlled trial
Source: BMC Med Educ. 2019 Dec 4;19:454. doi: 10.1186/s12909-019-1887-1 (PMC6894350; doi:10.1186/s12909-019-1887-1)
Supplement: Supplementary file 2 — Additional file 2. VP case example: Traumatology case 2. [file 12909_2019_1887_MOESM2_ESM.pdf]

Traumatology Case Nr. 2

CT Scan Evaluation

Which of the following statements are false?

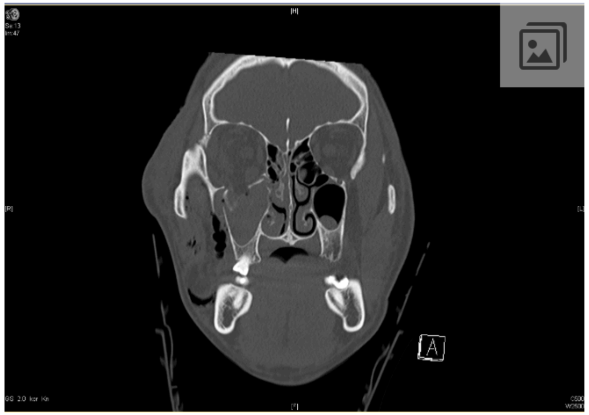

1. The depicted image shows a sagittal reconstruction ☐
2. The central facial buttress is fractured ☐
3. The CT demonstrates blood in the right maxillary sinus ☐
4. Subcutaneous emphysema appear as hyperdense areas inside the soft tissues ☑

Subcutaneous emphysema is readily visible on CT scans, with pockets of gas seen as extremely dark low (air) attenuation areas in the subcutaneous space.

5. The CT demonstrates a displaced fracture of the zygoma ☑

In zygomaticomaxillary fractures the following fracture components are generally identified: fracture of the zygomatic arch, fractures of the inferior orbital rim and diastasis of the frontozygomatic suture.

Check answers | Show responses | Reset
