# Supplementary material for: Virtual patients versus small-group teaching in the training of oral and maxillofacial surgery: a randomized controlled trial
Source: BMC Med Educ. 2019 Dec 4;19:454. doi: 10.1186/s12909-019-1887-1 (PMC6894350; doi:10.1186/s12909-019-1887-1)
Supplement: Supplementary file 3 — Additional file 3. VP case example: Traumatology case 1. [file 12909_2019_1887_MOESM3_ESM.pdf]

Traumatology

Craniofacial examination

Clinical craniofacial examination plays an essential role in assessing the damage caused by a traumatic event. When performing the examination a structured approach is recommended to assure best practice.

Begin by assessing the function of the cranial nerves:

a. Pupillary light reflex: Equally dilated pupils, direct and consensual response.

Pathology (G)

b. Clarity of Vision (G):

The ability of the patient to count fingers and recognize colors should be assessed.

Pathology(G)

c. Ocular motility:

Disturbances of ocular motility following

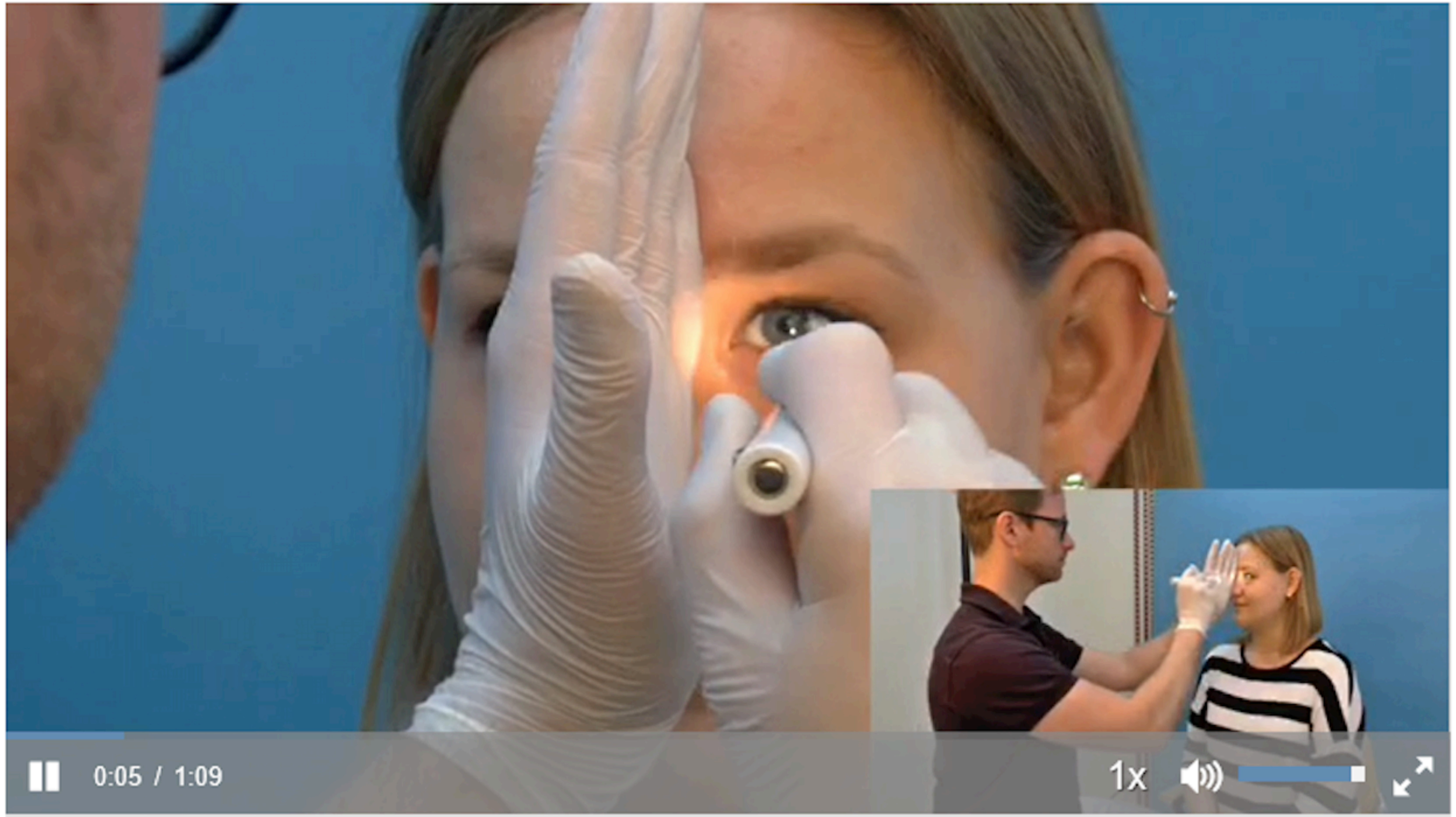

Demonstration of a structured craniofacial examination
